# High-Field Nonlinear Terahertz Conductivities of Iron Ultrathin Films

**DOI:** 10.3390/nano15181386

**Published:** 2025-09-09

**Authors:** Lewen Zhu, Zhiqiang Lan, Yingyu Guo, Danni Li, Lin Xi, Huiping Zhang, Zuanming Jin

**Affiliations:** 1 Shanghai Key Lab of Modern Optical System, Terahertz Spectrum and Imaging Technology Cooperative Innovation Center, Terahertz Technology Innovation Research Institute, University of Shanghai for Science and Technology, Shanghai 200093, China; 2235060530@st.usst.edu.cn (L.Z.);; 2 Anjieli Electronic Technology (Suzhou) Co., Ltd., No. 188, Lushan Road, Suzhou 215000, China; 3 Shanghai Institute of Intelligent Science and Technology, Tongji University, Shanghai 200092, China

**Keywords:** Fe thin films, high terahertz field, nonlinear conductivity, Drude–Smith model

## Abstract

The electronic transport behavior in ferromagnetic thin films critically dictates the functionality and efficiency of devices in spintronics and modern materials science. This work characterizes terahertz (THz) responses and nonlinear conductivities of Fe ultrathin films under high-field THz excitation. We demonstrated that different nonlinearities are present for two different thickness samples. For a 2 nm thick Fe film, as the peak THz electric field was increased to 369 kV/cm, the THz transmittance of Fe films generally decreased. However, for the 4 nm thick Fe film, the THz transmittance is almost field strength independent. This result is correlated with the conductivity variations induced by carrier transport processes. The real part of the complex conductivity for the 2 nm thick film increased significantly with the THz electric field, while the 4 nm thick film showed negligible dependence. In addition, we extracted the frequency-domain complex conductivity of the Fe thin films and used the Drude or Drude–Smith model to explain the distinct behaviors between the two thickness samples under intense THz fields, mainly associated with the surface morphology. This work aims to elucidate the transport properties of Fe films in the THz frequency range. Our findings lay a crucial foundation for the design and development of future high-performance THz spintronic functional devices.

## 1. Introduction

Ferromagnetic thin films, particularly iron (Fe) films, have attracted considerable attention in condensed matter physics and materials science owing to their unique electronic, magnetic, and transport properties, rendering them crucial for spintronic devices, ultrafast optoelectronics, and terahertz (THz) technology [1,2,3,4]. Fe exhibits tunable magnetic anisotropy, thereby enabling precise modulation of its physical properties via external stimuli such as electric fields, strain, or optical pulses [5,6,7,8,9,10]. In recent years, the nonlinear optical properties, ultrafast magnetization dynamics, and inverse Faraday effect of Fe thin films have also been investigated [11,12,13]. In addition, Fe films and Fe-based heterostructures show promising candidate materials for THz emitters and modulators, where understanding their ultrafast carrier dynamics and THz response is critical [3,14,15,16,17].

The THz electromagnetic field exhibits unique capabilities in probing intra-band electron transitions, lattice vibrations, and molecular motions—such as low-frequency bond vibrations and hydrogen bond dynamics [18,19,20,21]. Coupled with its non-destructive, label-free characteristics and a broadband range that aligns with intermolecular interaction energies, THz radiation emerges as an ideal tool for investigating material properties [22,23,24,25,26,27]. THz time-domain spectroscopy (THz-TDS), via coherent measurement, allows simultaneous measurement of the amplitude and phase of THz transmission, enabling accurate extraction of conductivity spectra and carrier’s parameters, for example, carrier scattering time constant and plasma frequency [28,29,30,31]. Complex conductivity responses in the THz spectral range are critical for unraveling the carrier transport mechanisms and underpinning device optimization. This technique has been successfully applied to study ferromagnetic materials, showing that thickness alters carrier localization and backscattering in Fe films [32,33]. Huang et al. conducted experimental studies on Fe films of different thicknesses using THz-TDS. Their findings revealed the influence of magnetization states on complex conductivity and demonstrated the promise of non-invasive THz probing methodology for studying spintronic materials at ultrafast timescales [34]. Nevertheless, experimental nonlinear THz conductivity response of Fe thin films—particularly the carrier’s transport parameters—remains to be investigated.

In this work, we measure the THz transmission and conductivity properties of 2 nm and 4 nm thick Fe thin films excited by an intense THz pulse. The Drude model or the Drude–Smith model was utilized to conduct an analysis of the complex conductivity spectra. Based on this, we investigated the distinct responses of the two Fe thin films with different thicknesses under high-field THz pulse excitation. This study aims to unravel the ultrafast carrier transport mechanisms in Fe thin films, thereby establishing a foundational understanding to guide the design of Fe-based THz devices and spintronic applications.

## 2. Materials and Methods

### 2.1. Sample Preparation

**Figure 1:**
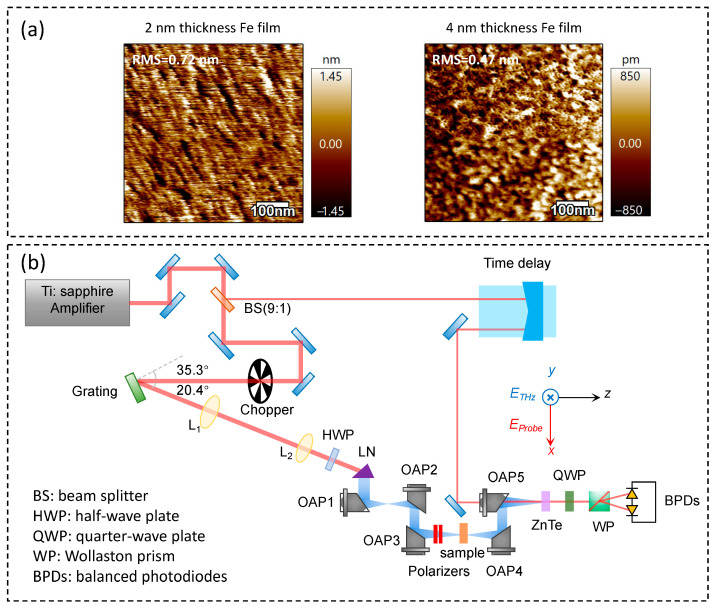
(**a**) AFM images of 2 nm and 4 nm thick Fe thin films grown on MgO substrate. (**b**) Schematic diagram of the THz-TDS setup with the tilted-pump-pulse-front technique.

Using magnetron sputtering, we fabricated 2 and 4 nm thick Fe films on single-crystalline MgO (001) substrates. All Fe films were capped with a 2 nm thick SiO_2_ layer for protection. The sputtering chamber was maintained at a base pressure of 5 × 10^−5^ Pa. Argon was used as the sputtering gas. Pre-sputtering was performed to clean the impurities from the Fe target surface, and the sputtering power was controlled by adjusting the current to tailor the film growth kinetics, ensuring the formation of Fe thin films on the MgO substrate [35]. Atomic force microscopy (AFM) images depicting the surface morphology of 2 nm and 4 nm Fe films are presented in Figure 1a. It can be seen significant difference in surface inhomogeneities and defects between 2 nm and 4 nm films. The surface roughness of the 2 nm and 4 nm thick films is determined to be 0.72 and 0.47 nm root mean square roughness (RMS), respectively.

### 2.2. Experimental Setup

The THz-TDS measurement is depicted in Figure 1b. Intense THz radiation was emitted from a lithium niobate crystal (LiNbO_3_, LN) via the tilted-pump-pulse-front technique using femtosecond laser pulses. An amplified Ti: sapphire laser amplifier (Coherent Legend Elite series) was employed to produce laser pulses with the following parameters. The central wavelength is 800 nm, the pump power is about 825 mW (at a 10 mm beam diameter), the repetition rate is 1 kHz, and the pulse duration is ~120 fs. The pump pulse was first diffracted by a grating, then passed through two cylindrical lenses, and, finally, directed onto the LN crystal. The LN crystal was grown using the Czochralski method. To minimize Fresnel losses, both the input and output surfaces of the crystal were coated with anti-reflection layers. The THz pulses, generated via the second-order nonlinear optical effect, were collimated and focused using gold-coated off-axis parabolic (OAP) mirrors. A pair of wire-grid polarizers served to control the THz pulses’ intensity. The electric field of THz pulses can be evaluated by using the following equation [36,37,38,39]:

(1) $$E_{\mathrm{peak}}=\sqrt{\frac{Z_{0}W}{\pi \alpha^{2}\int A^{2}(t)\mathrm{dt}}}$$

where THz peak field is *E_peak_* (V/cm), *Z*_0_ = 377 Ω is the impedance of free space, *W* stands for the energy value of the THz pulse (J), π*α*^2^ is the area of the THz beam, and *A*(*t*) is the temporal profile of a THz pulse. The average power of the THz pulse was measured at about 2.50 mW by a pyroelectric detector. The peak electric field of the THz pulse was estimated to be ~662 kV/cm at the focal position using Equation (1).

As shown in Figure 1b, a Cartesian coordinate system was used to define the optical path: the THz wave propagated along the z-axis, with normal incidence on the sample. The intense THz pulse interacted with the Fe thin film, inducing field-dependent changes in THz transmission. The transmitted THz signals were collected via off-axis parabolic mirrors (OAP4–OAP5) and measured through electro-optical sampling in a 0.5 mm thick ZnTe <110> crystal. The probe beam passed through a quarter-wave plate (QWP) and Wollaston prism (WP), which splits into orthogonal components for detection by a balanced photodetector. Outputs from the balanced photodetectors (BPDs), connected to a chopper-synchronized lock-in amplifier, enabled high-sensitivity temporal measurement of THz signals. A delay stage was employed to adjust the time delay between the THz and probe beams, enabling measurements of both the peak electric field and the temporal profile of the THz waveforms. In the experiment, the sample was positioned at the focal spot of the THz beam. We passed dry air through the entire system to remove water vapor absorption (relative humidity below 10%).

## 3. Results and Discussion

**Figure 2:**
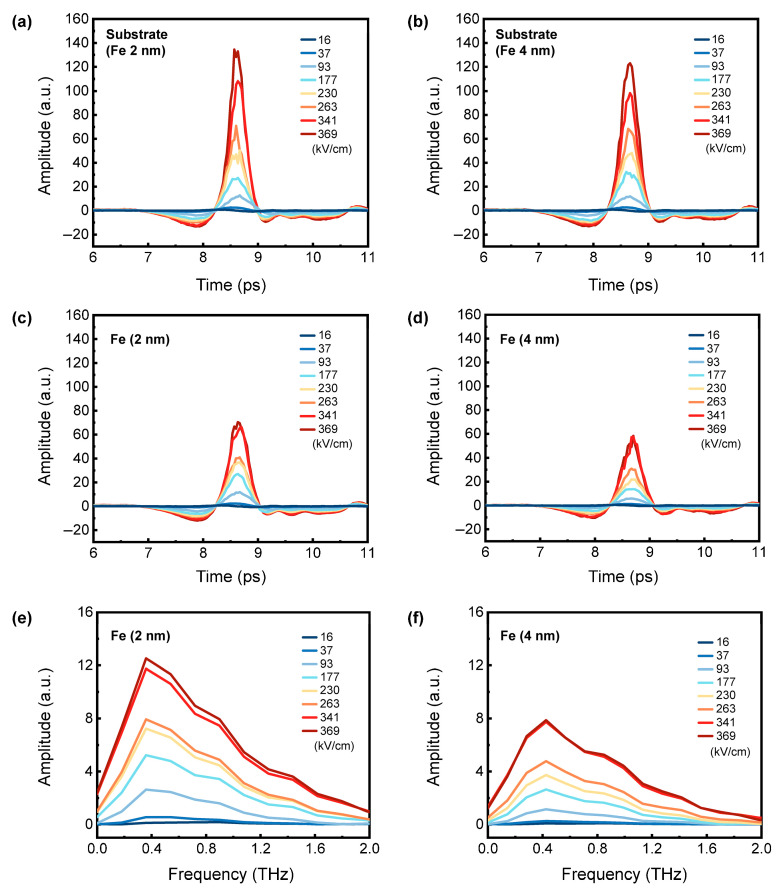
Time-domain THz transmitted signals through the MgO substrates used to grow Fe thin films with thicknesses of (**a**) 2 nm and (**b**) 4 nm, respectively, under different THz electric fields. THz electric fields transmitted through (**c**) 2 nm and (**d**) 4 nm thick Fe thin films on MgO substrates under different THz electric fields. Frequency-domain THz transmission spectra of (**e**) 2 nm and (**f**) 4 nm thick Fe thin films under different THz electric fields.

Figure 2a and Figure 2b show the time-domain THz transmitted signals *E_sub_*(*t*) through the MgO substrates, which are used to grow Fe thin films with thicknesses of 2 nm and 4 nm, respectively. Figure 2c,d present the time-domain THz signals *E_sam_*_+*sub*_(*t*) transmitted through the 2 nm thick and 4 nm thick Fe thin films on MgO substrates under different THz electric fields. Applying the Fourier transform to time-domain signals yielded the frequency-dependent THz signals. The corresponding frequency-domain signals *E_sam_*_+*sub*_(*ω*) of Fe films are shown in Figure 2e,f.

**Figure 3:**
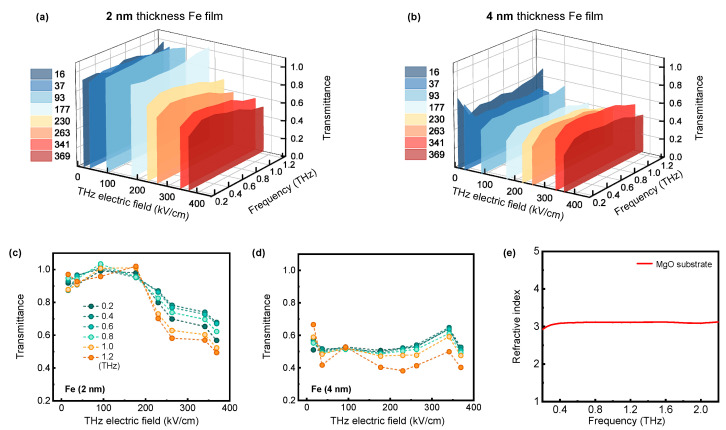
Dependence on THz frequency of the transmittances for (**a**) 2 nm and (**b**) 4 nm thick Fe films across the 0.2–1.2 THz range under different THz peak electric fields. THz transmittance of (**c**) 2 nm and (**d**) 4 nm thick Fe films as a function of THz peak electric field strength at several selected frequencies from 0.3 to 1.2 THz. The circular symbols of the curves in (**d**) are consistent with those in (**c**). (**e**) MgO substrate’s refractive index spectrum measured at room temperature.

Figure 3a and Figure 3b depict the THz transmittance spectra of 2 nm and 4 nm thick Fe films as a function of THz frequency under different peak THz electric fields from 16 to 369 kV/cm, respectively. A key observation is that two Fe thin films with different thicknesses exhibit distinct transmittance responses to the increasing peak THz electric fields. The 2 nm thick Fe film shows a significant decrease in THz transmittance as the THz electric field increases. A reduction in the THz transmission is indicative of an increase in the conductivity. In contrast, the 4 nm thick Fe film maintains a nearly constant THz transmittance. Figure 3c and Figure 3d show the THz transmittance of 2 nm and 4 nm thick Fe films as functions of peak THz electric field measured at different THz frequencies, respectively. In Figure 3c, for the 2 nm Fe film, as the THz peak electric field varies from 16 to 369 kV/cm, the transmittances of THz pulses passing show nonlinear responses. The THz transmittance decreases from approximately ~0.9–1.0 to ~0.6–0.7 as the THz electric field rises from 16 to 369 kV/cm. In Figure 3d, for the 4 nm Fe film, as the THz electric field varies from 16 to 369 kV/cm, the THz transmittance fluctuates approximately ~0.4–0.6. Under the lowest THz electric fields, the optical conductivity of the 4 nm Fe film exceeds that of the thinner sample. When the 2 nm Fe film is exposed to the strongest THz field magnitude, the THz transmittance values of both the 2 nm and 4 nm thick Fe films become closer.

Owing to the conductivity of Fe films, the THz electric field generates a time-varying current within them. This induces attenuation of the THz wave, which in turn brings about a decrease in transmittance. The THz complex conductivity spectra of 2 nm and 4 nm Fe films with different THz peak electric fields can be derived from the transmittance spectra presented in Figure 3a,b, using the following relationship [40,41]:

(2) $$\frac{{\tilde{E}}_{\mathrm{sam}+\mathrm{sub}}(\omega )}{{\tilde{E}}_{sub}(\omega )}=\frac{1+n_{sub}}{1+n_{sub}+Z_{0}\tilde{\sigma }(\omega )d_{sam}}exp[\frac{i\omega }{c}(n_{sub}-1)\Delta L]$$

where free-space impedance $Z_{0}\;=\;377\;\Omega$, $\tilde{\sigma }(\omega )$ is the complex conductivity of the Fe film, *d_sam_* is the Fe film’s thickness, and *ω* = 2π*f* (with f being the THz frequency). We measure the MgO substrate’s refractive index *n_sub_* by our THz-TDS. As shown in Figure 3e, the average *n_sub_* is approximately 3.1 in the range of 0.2–2 THz, which is consistent with the value in the literature [42]. In Equation (2), ΔL is the difference in thickness between the substrate on which the samples were grown and the reference substrate. For 2 nm and 4 nm thickness Fe films, ΔL are +1.0 μm and −5.0 μm, respectively. The ΔL values were experimentally measured using a high-precision digital micrometer. $\tilde{\sigma }(\omega )=\sigma_{1}(\omega )+i\sigma_{2}(\omega )$ is calculated using Equation (2) at frequencies between 0.3 and 1.2 THz. We have corrected the conductivity deviation arising from the phase error, $\frac{\omega }{c}(n_{sub}-1)\Delta L$.

**Figure 4:**
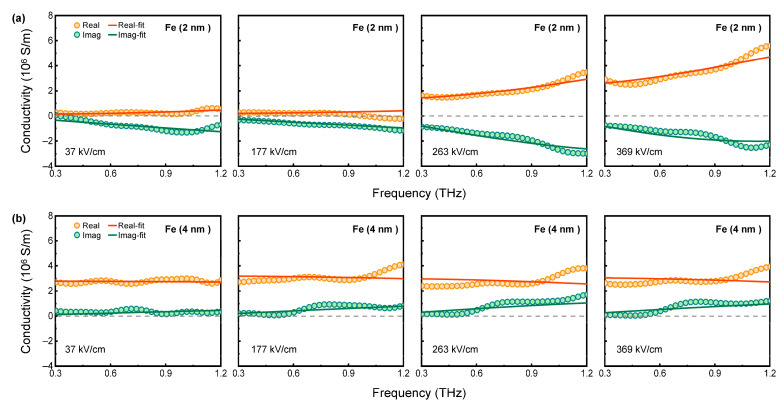
The real parts (orange circles) and imaginary parts (green circles) of the conductivity spectra in the THz frequency range for Fe films with thicknesses of (**a**) 2 nm and (**b**) 4 nm under different peak THz electric fields. The solid curves represent the fitting by the Drude–Smith or Drude model.

Figure 4a and Figure 4b present the THz complex conductivities of Fe films with 2 nm and 4 nm thicknesses, respectively. Orange circles and green circles denote the real parts and imaginary parts of complex THz conductivity, respectively. Within the 0.3–1.2 THz range, the two Fe films exhibit distinct conductivity behaviors dependent on the THz peak electric field. For the 2 nm thick Fe film, the real part of the conductivity $\sigma_{1}(\omega )$ is positive and increases significantly as the THz peak electric field increases. When the applied electric field remains below 177 kV/cm, the film exhibits a transmittance close to ~100% and negligible conductivity $\sigma_{1}(\omega )$, consistent with insulating behavior. With increasing THz field strength, the emergence of positive conductivity $\sigma_{1}(\omega )$ signifies the material’s transition from an insulating state to a conductive state. In contrast, the $\sigma_{1}(\omega )$ for the 4 nm thick Fe film shows negligible dependence on THz field intensity, remaining nearly constant within the entire measured THz electric field range. The imaginary part of the conductivity $\sigma_{2}(\omega )$ for 2 nm thick Fe film shows almost negative values within the 0.3–1.2 THz range. As the peak THz electric field increases, the negative value of $\sigma_{2}(\omega )$ increases significantly. In contrast, the $\sigma_{2}(\omega )$ of the 4 nm thick Fe film is almost positive, which also depends on the incident THz electric field intensity.

Analysis of the measured THz conductivity spectra using the Drude–Smith (DS) model provided a deeper investigation of carrier transport in Fe films within the THz frequency range. The DS model is commonly used to describe systems where carriers experience backscattering or localization effects. The DS model has been validated as effective in analyzing THz conductivity of magnetic metallic structures [43]. The formula of the DS model is expressed as follows [44,45,46,47]:

(3) $$\tilde{\sigma }(\omega )=\frac{\varepsilon_{0}\omega_{p}^{2}\tau }{1-i\omega \tau }[1+\frac{c}{1-i\omega \tau }]$$

where *ε*_0_ is the vacuum permittivity, *ω_p_* represents the plasma frequency, *τ* denotes the momentum scattering time, and *c* (ranging from −1 to 0) is a phenomenological coefficient quantifying carrier backscattering or localization degree. DC conductivity is $\sigma_{dc}=\varepsilon_{0}\omega_{p}^{2}\tau (1+c)$. The parameter *c* is associated with the “memory effect” of carrier velocity. As shown in Figure 4a,b, the orange solid lines are fits of the real-part conductivity, and the green solid lines are fits of the imaginary-part conductivity. The model displays reasonable agreement with the experimental results.

**Figure 5:**
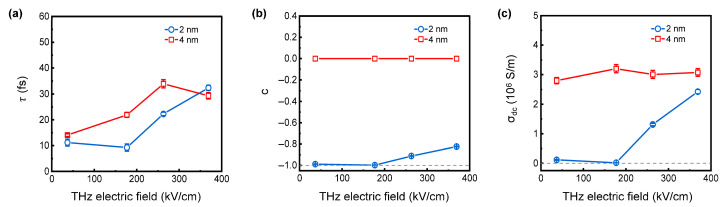
The Drude–Smith fitting parameters, including (**a**) momentum scattering time *τ*, (**b**) backscattering coefficient *c*, and (**c**) DC conductivity *σ_dc_*, for Fe films with thicknesses of 2 nm and 4 nm as functions of THz peak electric fields at room temperature.

We extract *τ*, *c*, and *σ_dc_* for 2 nm and 4 nm thick Fe films, as summarized in Figure 5. The momentum scattering time *τ* presented in Figure 5a shows that the 2 nm thick and 4 nm thick Fe films exhibit a dependence of the momentum scattering time on the THz field strength. At the lowest THz field strength, *τ* is 11.16 ± 1.34 fs for the 2 nm thick Fe film, which is shorter than *τ =* 14.04 ± 0.50 fs for the 4 nm thick Fe film. This is consistent with the results from Krewer et al. [33], in which a significant reduction in momentum relaxation time with decreasing Fe film thickness was found. This is due to the inhibited surface scattering of long mean-free-path electrons. It is important to see that the momentum relaxation time increases with the incident THz pulse field intensity.

As shown in Figure 5b, for the 2 nm thick Fe film, the *c* is close to −1 under the peak THz electric field ranging from 37 to 177 kV/cm, which corresponds to a fully backscattered state. As the THz peak electric field is further increased, *c* increases accordingly. In contrast, for the 4 nm thick Fe film, *c* ≈ 0, the DS model is reduced to the Drude model. Our results indicate different degrees of electron localization in two films, which is primarily due to the differences in surface scattering effect, as confirmed by the AFM image.

Figure 5c presents the *σ_dc_* for the 2 nm thick and 4 nm thick Fe films. For the 2 nm thick Fe film, its *σ_dc_* remains nearly 0 S/m under the THz peak electric field ranging from 37 to 177 kV/cm. As the THz peak electric field is further enhanced, the *σ_dc_* increases accordingly. In contrast, the *σ_dc_* of the 4 nm thick Fe film is almost independent of the THz electric field strength, *σ_dc_* ≈ 3.0 × 10^6^ S/m. The divergent THz electric field-dependent conductivity behaviors between two Fe films arise from distinct carrier transport mechanisms, rooted in their surface microstructural differences.

In the THz range, the photon energy amounts to only several millielectron volts, which is insufficient to induce electronic transitions. Consequently, the induced THz field optically excites electrons in iron thin films, with the electrons gaining energy in the course of this excitation. The kinetic energy of these excited electrons is then passed to other electrons and the lattice through electron–electron and electron–phonon coupling, respectively.

For the 2 nm thick Fe film, as shown in Figure 1a, high defect density and discontinuous nano-film are observed. The conductivity model involves the inter-grain tunneling affected by surface defects. At low THz electric fields, the defects form strong localized states that limit carrier transport—this leads to extremely low conductivity, and high THz transmittance. When the THz field exceeds 177 kV/cm, the field intensity becomes strong enough to trigger carrier ionization. Then, the localized carriers become free carriers. Consequently, this drives a rapid rise in real conductivity. In contrast, the 4 nm thick Fe film exhibits fewer defects and more stable grain boundaries. Thus, the conductivity model involves metallic conduction. The DC conductivity is nearly insensitive to the THz field intensity. Our observations align with previous reports on how defect and grain boundary scattering modulate THz-driven carrier transport [48,49,50].

## 4. Conclusions

To summarize, the THz transmission properties of 2 nm and 4 nm thick Fe thin films under varying peak THz electric fields were characterized using THz-TDS, with the THz complex conductivity spectra extracted. Fitting the THz conductivity of the 4 nm thick Fe thin film is achieved with the Drude model, while the Drude–Smith model yields a good fit for the THz conductivity of the 2 nm thick Fe thin film, enabling quantitative determination of microscopic carrier transport parameters. The effect of THz electric fields on key transport parameters—including dc conductivity, carrier momentum scattering time, and back-scattering coefficient—was quantitatively investigated in two Fe thin films. An increase in DC conductivity was observed as the thickness of the Fe film increased. In addition, it was observed that, under varying THz electric fields, thicker Fe films exhibit distinct modulation trends in these parameters relative to thinner counterparts, revealing the combined modulation of surface morphology and electrical field on carrier transport. The THz spectral and transport properties of Fe thin films elucidated herein hold implications for the design of ferromagnetic thin-film-based THz functional devices.

## Data Availability

The data presented in this study are available on request from the corresponding author.
